# A systematic review of online depression screening tools for use in the South African context

**DOI:** 10.4102/sajpsychiatry.v25i0.1373

**Published:** 2019-11-12

**Authors:** Tasneem Hassem, Sumaya Laher

**Affiliations:** 1 Department of Psychology, University of the Witwatersrand, Johannesburg, South Africa

**Keywords:** BDI-II, CES-D, depression, major depressive disorder, PHQ-9, screening tool

## Abstract

**Background:**

According to the World Health Organization, the alarming increase in rates of depression globally has become a serious concern. In 2010, the prevalence rate of depression in South Africa was 4.6%. Given the context of South Africa where the majority of the population have limited access to healthcare facilities and 59.3% of the population have access to the Internet, an online depression screening tool would have much to offer.

**Objective:**

To determine whether online depression screening tools would be suitable for use in South Africa.

**Methods:**

This study presents a systematic review of online depression screening tools to determine whether one would be suitable for use in South Africa. Articles were accessed from seven electronic databases from 1970 to 2018. All articles included in the review were critically appraised.

**Results:**

A total of 17 articles met the inclusion criteria. From the results, there was only one screening tool available on an open access platform for use by the general population. The most common depression online screening tools were the Beck Depression Inventory-II (BDI-II), the Center for Epidemiology Studies Depression Scale (CES-D) and the Patient Health Questionnaire (PHQ-9). It was also evident that there were negligible differences in the psychometric properties of online versus paper versions of the online screening tools. Furthermore, there were very few studies that considered the African or South African population and no online screening tools for major depressive disorder (MDD) developed in these contexts.

**Conclusion:**

There appears to be a need for a depression screening tool to be adapted for online usage in South Africa. It is recommended that the online screening tool should be adapted from the three commonly used online depression screening tools: PHQ-9, CES-D and BDI-II.

## Introduction

A worldwide increase in depression prevalence rates by more than 18% from 2005 to 2015 has listed depression as a leading cause of disability and ill health.^1^ According to the global burden of disease study in 2010, 5.0% of the southern sub-Saharan African population was diagnosed as having major depressive disorder (MDD)^2^ In South Africa, the MDD prevalence rate in 2010 was 4.6%.^2^ The majority of depression screenings are first made in primary care facilities, where accurate diagnoses of depression in patients only occur in less than 50.0% of cases.^3^ This inaccuracy is often attributed to the lack of resources available in these facilities, time constraints, lack of training as well as screening tool bias.^4^ Primary healthcare providers often want to use screening tools that require the least amount of training and time to administer and interpret.^5^

The majority of the paper-based depression screening tools are based on the Diagnostic and Statistical Manual of Mental Disorders (DSM), 4th Edition (DSM-IV) or DSM, 4th Edition, Text Revision (DSM-IV-TR) classification of MDD.^5,6^ This classification was revised in the DSM 5th Edition (DSM 5),^7^ where the categories of a single and recurrent depressive episode, as well as the symptoms of bereavement, were removed. Furthermore, the DSM criteria for depression are often criticised for being based on a Western set of cultural assumptions. These assumptions include the autonomy and uniqueness of each individual, the focus on the intrapersonal rather than interpersonal symptoms and the emphasis on emotional symptoms as a classification for depression. These Western cultural norms are not universal as various cultures view individuals as being interdependent; and the mind and body are not viewed as distinct entities but rather as mutually constitutive.^8^ The DSM classification is based on a dichotomous approach when it comes to MDD diagnoses, but this approach is unclear. As a result, depression can either be overdiagnosed or underdiagnosed and depression diagnostic tools should be utilised with extreme caution in non-Western-based societies.

Hence, the applicability of the tools designed using these criteria must be explored.^3^ Illness presentation in African cultures is bound to traditional religious beliefs, social relations as well as cosmology.^9^ While spirituality and culture are relevant across the diverse spectrum of the South African population, screening tools for depression have not been adapted to account for these unique cultural and spiritual presentations of depression.

South African research on MDD screening has considered the paper versions of the Patient Health Questionnaire 9 (PHQ-9), Center for Epidemiological Studies Depression Scale (CES-D) and Beck Depression Inventory II (BDI-II).^10,11,12,13^ Smit et al.^10^ reported lower sensitivity and specificity scores for the South African HIV-positive sample when compared to the pooled analysis for the CES-D. Kagee et al.^11^ found that the BDI-II was a reliable screening tool amongst HIV-positive patients in South Africa. Baron et al.^12^ used the isiZulu, isiXhosa and Afrikaans versions of the 10 item CES-D (CES-D 10) with a sample from the general population and reported a sensitivity of 71.4%, with 72.6% of individuals in the sample being correctly identified.

Makhubela et al.^13^ reported good reliability as well as good convergent and discriminant validity for the BDI-II in the general population. There have been no studies considering the online screening of MDD in South Africa.

Using the Internet to screen for a psychological disorder is becoming more common. With the increasing growth in Internet usage, it has become the first source for individuals to search for information.^14^ Information found on the Internet with regard to medical conditions is often updated with expert information, making it the largest medical library worldwide.^15^ The benefits of accessing medical information include the convenience, expert information that is accessed at little or no cost and privacy. Stigma against mental illness is still very prevalent in societies where people with depression are viewed as undesirable to be around as they are seen to be responsible for their own condition. Individuals fear to seek professional help because of the response they will get from community members.^16^ The Internet offers protection from such stigma.

While there are multiple benefits of using the Internet, the wealth of information is often overwhelming for many. Morahan-Martin^15^ conducted a Google search using the phrase ‘mental disorder test’. The results indicated 244 000 pages were available. The researcher’s personal search for this study using the phrase ‘depression screening tool’ yielded 170 000 pages of information on a Google search. These numbers suggest that there is a great demand for such tests by the general public. According to the General Household Survey 2016, 59.3% of households in South Africa had at least one member who had access to the Internet and 53.9% of South Africans had mobile Internet access.^17^ These figures suggest the possibility of online screening tools providing access to information in South Africa where mental health resources are limited and often inaccessible.^18^

In a scoping review and evaluation of 32 web-based intervention programmes for depression,^19^ the authors found that the majority of the programmes targeted an adult population (*n* = 19), while only two studies had a specific target population. Users of these programmes were required to complete a depression assessment either prior to registration, independent of the programme or during the programme, where results were received immediately upon completion. Of the 32 programmes, the authors report only 17 programmes to have used a validated depression screening tool such as the PHQ-9, BDI or the CES-D. However, it has not been stated if these tools were validated for online usage.

Concerns have been raised with regard to the effectiveness of online tests.^20^ Buchanan asserts that the psychometric properties of online tests differ when compared to pencil and paper versions of the test; therefore, these properties must be considered, despite the online version of the test being a direct translation of the pen-and-paper instrument.^20^ Very few of the psychological tests available were developed by professionals in the field therefore do not have established psychometric properties.^20^

Therefore, this study used the method of systematic review to establish if there were any appropriate online depression screening tools for use in the South African context. Hence, the specific questions for this study were:

- Which MDD screening tools are available online?
- What are the psychometric properties of these screening tools?
- Have any of these screening tools been used in the South African context?
- Which tool(s) can be adapted for the South African population if none have been used or adapted before?

## Methods

### Research design

A qualitative systematic review was the chosen method for this study, as a qualitative analysis was conducted on both quantitative and qualitative studies, which were included in this study.^21^ The eight-stage procedure for conducting systematic reviews recommended by Uman^22^ was followed. Stages 1–4 required the researcher to formulate the review questions, define inclusion and exclusion criteria, and develop a search strategy and, lastly, to select studies. During stages 5 and 6, the researcher needed to extract the data from included studies and critically appraise the included studies. Finally, during stages 7 and 8, the researcher was required to analyse and interpret the extracted data and disseminate the findings.

### Search process

The steps outlined in the Preferred Reporting Items for Systematic Reviews and Meta-Analyses (PRISMA) statement were followed for data collection.^23^ Articles were accessed from the Academic Search Complete, EBSCO Host: Psychology and Behavioural Science Collection, Sabinet, Academic Search Premier, PsychInfo, ProQuest Psychology journals and the PubMed electronic databases. These databases were selected as they provide international and African-focused multi- and interdisciplinary scholarly literature. Reference lists of selected articles were screened for any additional eligible articles. The following keywords were used: ‘online depression screening tools’, ‘Internet depression screening tools’, ‘using the Internet to screen for depression’, ‘web-based depression screening tools’, ‘web-based depression assessments’, ‘screening for depression on the Internet’ as well as ‘depression assessments on the Internet’. All articles’ search results were saved to Zotero (a referencing software). Articles were screened using three phases: title screening, abstract screening and full-text screening. Article titles and abstracts were screened by the first author as well as an independent researcher.

### Study eligibility

To be included in the sample, the following inclusion criteria were used: (1) articles needed to be written in English; (2) only articles published from 1970 to 2017 were considered as the first large-scale online testing and interpretation of psychological assessments occurred in the early 1970s;^24^ (3) the study must be conducted on adults from the general public (18 years and above); (4) the study must contain a description of the MDD screening instrument that must have been specifically designed or adapted for an online environment. Articles were excluded if the screening tool was used on patient (including medical and psychiatric) samples as this study focused on reviewing articles on a screening tool that could be used on the general population, not on those already diagnosed with MDD. Articles where the depression screening instrument was combined with another screening instrument were also excluded as psychometric properties reported were unclear for subscales of the tools. Grey literature was also excluded from the search as the authors deemed peer-reviewed research to be more rigorous.

### Data analysis, extraction and quality assessment

Data was analysed using content analysis, which proceeded in three phases.^25^ In the first phase units of analysis were selected, such as the instruments description section, sample section and the results section where instruments were validated. The second phase used an inductive approach where data was organised in terms of free coding, creating categories and abstraction. Lastly, data was reported in terms of the codes determined in phase 2. Included articles were assessed using the criteria proposed in the Critical Appraisal Skills Programme (CASP).^26^ Quantitative studies were scored out of 11 points, where a score between 11–8 was considered strong, 7–4 was considered moderate and 3–0 was considered weak. All studies received a score of 9, 10 or 11, with the exception of the study by Harvey et al.^27^ This qualitative study received a score of 5 out of 6, which represented a strong appraisal score^28^ (see Table 1).

**TABLE 1:** Descriptive data of included articles.

Authors	Type of study				Sample demographic		Sample age		Sample gender		Critical appraisal score
	Intervention	Comparison of paper versus Internet test	Internet test validation	Other	Size	Country	Range	Mean	Female (%)	Male (%)	
Lin et al.^39^ Lin et al.^40^	-	-	X	-	579	Taiwan	18–52	26.5	72.7	27.3	11
Donker et al.^38^	-	-	X	-	502	Netherlands	18–80	43	57	43	11
Harvey et al.^27^	-	-	-	X Association study	3401 of which 1161 completed depression screening tool	UK telecommunications company	Not provided	Not provided	Not provided	Not provided	5
Krog et al.^28^	-	-	-	X Qualitative study	9 general practitioners	Denmark	44–67	Not provided	3	6	5 (total of 6)
Leykin et al.^36^ Gill et al.^41^	-	-	X	-	24965 Monthly screening: 1371	United Kingdom (*n* = 384), followed by India (*n* = 244) and South Africa (*n* = 150); 15.2% of participants reported being born in a country other than the country of their current residence (consenting for the follow-up)	18–92	32.1	63.8	31.7	9
Spek et al.^29^	-	X	-	-	407	-	Not provided	55	64	34	11
Williams et al.^32^	-	-	-	X Feasibility study	972	US college students Massachusetts	Not Provided	71	29	9
Garlow et al.^43^	X	-	-	-	729	America	College students	Not provided	71.7	28.3	10
Jeong Youn et al.^33^	-	-	-	X Feasibility study	259	Five US colleges (California, Massachusetts and Pennsylvania	Not provided	77.4	22.6	9
Holländare et al.^30^	-	X	-	-	87	Sweden	20–72	41.1	65.5	34.5	11
Herrero et al.^31^	-	X	-	-	530	Open University of Catalonia	-	29.1	66	44	10
Monero et al.^42^	-	-	-	X Association study	215	College students in the two US universities	18–20	18.8	54	46	11
Moreno et al.^44^	-	-	-	X Association study	273	University students (USA)	Not provided	18.9	58.4	41.6	10
Du et al.^35^	-	-	X	230 at time 1 150 at time 2	University students in China	18–25	19.93	43.5	56.6	11
Lee et al.^34^	-	-	-	X Association study	6068	University students in Korea	Undergraduate: 18–30 Graduates: 20–47	Undergraduates: 20.3 Graduates: 26.4	23.9	76.1	10
Lup et al.^37^	-	-	-	X Association study	117	Facebook users	18–29	24.8	84	16	9
Liu et al.^45^	-	-	-	X Feasibility study	4709	Chinese literate individual	18 and older	30.2	61.8	38.2	10

Note: X represents the type of study indicated.

### Ethical consideration

Articles obtained for analysis in the review were all available in the public domain; as a result no special ethical considerations were required.

### Results

**FIGURE 1:**
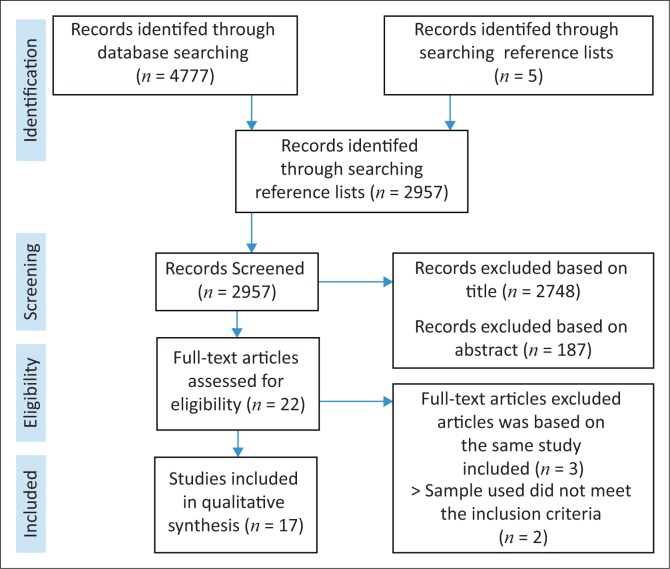
Preferred Reporting Items for Systematic Reviews and Meta-Analyses flow diagram of article inclusion and exclusion.

Through the various database searches and additional reference list searches, a total of 4777 articles were identified. After removing duplicates, 2957 article titles were screened. The total number of articles excluded based on titles and abstracts amounted to 2748 and 187, respectively. Seventeen articles were identified for possible inclusion in the study. As such, all 17 articles were used for the review (see Figure 1).

### Description of included articles

Table 1 provides a brief descriptive overview of the articles. Of the 17 studies, 5 articles looked at the association of depression and another psychological measure, 4 articles assessed the psychometric properties of an online screening tool for depression, 3 articles made a direct comparison of a paper versus Internet version of a depression screening tool,^29,30,31^ 3 articles were a feasibility study of an intervention that utilised a depression screening tool,^32,33^ 1 article was based on an intervention study and 1 article was a qualitative descriptive article. The qualitative descriptive article obtained data on the experience of an online depression screening tool from general practitioners.^33^

Sample sizes obtained in each study ranged from 87 to 24 965 individuals, with the exception of the qualitative study. The qualitative study conducted by Krog et al.^28^ interviewed nine general practitioners. The majority of the participants in the study samples were women,^15^ with the exception of the studies conducted by Krog et al.,^28^ Lee et al.,^34^ and Du et al.^35^ All study samples included participants over the age of 18 years, with an age range between 18 and 92 years. Samples recruited in the studies were representative of very specific target populations, with the majority of the studies recruiting US citizens. The only study open to a global sample was that by Leykin et al.,^36^ where anyone who had access to the Internet could participate. Of the sample obtained for a follow-up screening, the following countries were represented: the United Kingdom, India and South Africa (*n* = 150). No other studies were conducted in Africa or South Africa.

### Tools available online

The following screening tools were used in the studies: PHQ-9, CES-D, BDI-II, Edinburgh Depression Scale (EDS), Single Item Depression (SID) Scale (Dutch version), electronic Major Depressive Inventory (eMDI) (Dutch version), Major Depressive Episode (MDE) Screener, Internet-Based Self-Assessment Program for Depression (ISP-D), Kessler Psychological Distress Scale (K10) and the self-rating version of the Montgomery Asberg Depression Rating Scale (MADRS-S) (Table 2). The PHQ-9 was utilised by the majority (*n* = 7) of studies, followed by the CES-D (*n* = 3) and BDI-II (*n* = 3). The remainder of the depression screening tools were only utilised once by their respective studies.

**TABLE 2:** Description of online depression screening tools.

Authors	MDD tool used	Depression diagnosis criteria	Reliability information	Validity information
Lin et al.^39^ Lin et al.^40^	ISP-D	DSM-IV	Test retest (184 participants): 2 weeks: 0.830 2–4 weeks: 0.449 longer than 4 weeks: 0.499.	Sensitivity: 76.4%, specificity: 81.8% (55 participants)
Donker et al.^38^	SID (Dutch version) CES-D (Dutch version) K10 (Dutch version).	CES-D: previously validated depression scales (BDI).	CES-D: *α* = 0. 92 K10: *α* = 0.90 SID consists of only one item. Cronbach’s *α* could not be calculated.	Correlations among the three measures ranged from 0.68 of the SID with the CES-D and with the K10 (both *p* ˂ 0.001) to 0.84 of the CES-D with the K10 (*p* ˂ 0.001) SID: cut off score 5: sensitivity: 0.87; specificity: 0.51 CES-D: cut off score 22: sensitivity: 0.94; specificity: 0.62 K10: cut-off score 29, 31, 32: sensitivity: 0.69–0.81; specificity: 0.67–0.79.
Krog et al.^28^	eMDI (Dutch version)	ICD 10	Not Provided	No formal validity information provided. However, authors note that previous experience with the MDI paper made the eMDI easier to interpret and use. eMDI made process of documenting patient scores easier and efficient. The time taken to login to the MDI is relatively long.
Spek et al.^29^	EDS	-	The Internet-administered EDS has a good internal consistency: comparable to that of the paper and pencil EDS *α* = 0.87	The Internet-administered EDS correlated significantly with the Internet-administered BDI (*r* = 0.75; *p* < 0.001). The positive predictive values were comparable to those found in previous paper and pencil studies.
Leykin et al.^36^ Gill et al.^41^	MDI	DSM-IV	Not provided	No validity values provided. However, authors state that good agreement with PRIME-MD and with clinician-administered diagnostic interviews. References provided was checked and none were done using the online version. Authors also note this in limitations; however, they indicate that there is evidence that shows Internet versions of health questionnaires show few if any difference to paper versions.
Moreno et al.^44^	PHQ-9	DSM-IV	Not provided	Not provided
Harvey et al.^27^	PHQ-9	DSM-IV	Not provided	Not provided
Williams et al.^32^	PHQ-9	DSM-IV	Not provided	Description of the validity of the paper-based PHQ was provided
Moreno et al.^42^	PHQ-9	DSM-IV	Not provided	No formal validity information provided. However, authors note that the tool has been previously validated. References provided were checked and the validity information provided was not for the online tool
Du et al.^35^	PHQ-9 (Chinese version)	DSM-IV	Cronbach’s alpha: 0.8 two-week test retest: 0.78 item correlations: 0.54–0.69	Cut-off score of greater than or equal to 10 sensitivity: 0.74 and specificity = 0.85 Likelihood ratio of 5.08. No major difficulties in the administration. Students were satisfied with the scale; however, on the satisfaction rating comprehension was judged negatively
Garlow et al.^43^	PHQ-9	DSM-IV	Not provided	Not provided
Jeong Youn et al.^33^	PHQ-9	DSM-IV	Provide reference to the paper version psychometrics.	Provide reference to the paper version psychometrics.
Lee et al.^34^	BDI (Korean version)	DSM-IV	Report previous alphas and test–retest for the paper-based test for both psychiatric and non-psychiatric patients.	Not provided
Holländare et al.^30^	MADRS-S & BDI	BDI: DSM-IV MADRS-S: Comprehensive Psychopathological rating scale (1979 scale was developed)	Cronbach’s alpha levels for the MADRS-S for the online version of the test ranged from 0.73 to 0.81 and the paper version was 0.81. For the BDI-II, Cronbach’s alpha levels for the online version of the test ranged between 0.87 and 0.89 and the paper version was between 0.89 and 0.90.	Correlations between the Internet and paper versions of all MADRS-S items were significant. (*r* = 0.84 and 0.79 for suicide item) Correlation between the BDI-II total scores from the Internet administration and the paper administration was high, *r* = 0.89 and 0.80 for the suicidal item.
Herrero et al.^31^	CES-D	Previously validated tests (BDI)	CES-D (seven items) Scale virtually equalled as for both conditions; Internet *α* = 0.82 and paper *α* = 0.83.	Confirmatory factor analysis showed that both the Internet and paper version of the CES-D loaded in a single factor.
Lup et al.^37^	CES-D	Previously validated tests (BDI)	Report paper-based properties.	Report paper-based properties.
Liu et al.^45^	MDE (Chinese version)	-	Report paper-based properties.	Report paper-based properties.

ISP-D, Internet-Based Self-Assessment Program for Depression; CES-D, Center for Epidemiology Studies Depression; BDI, Beck Depression Inventory; DSM, Diagnostic and Statistical Manual; DSM-IV, Diagnostic and Statistical Manual of Mental Disorders, 4th Edition; PHQ, Patient Health Questionnaire; SID, Single Item Depression; MDE, major depressive episode.

### Psychometric properties of tools

Despite being used in the majority of studies, only one of the studies reported on the validity and reliability of the PHQ-9 tool. The study conducted by Du et al.^35^ utilised the Chinese version of the PHQ-9 and reported a Cronbach’s alpha score of 0.8, test–retest reliability for a 2-week period of 0.78 and inter-item correlations of 0.54–0.69. Three studies^32,33,42^ made mention of psychometric properties of the paper version of the PHQ-9 (see Table 2).

Of the three studies that utilised the CES-D, only one study did not report psychometric properties for the online version of the instrument (see Lup et al.).^37^ Herrero et al.^31^ showed that the reliability for the paper and online versions of the CES-D were virtually equal (0.83 and 0.82, respectively). A relatively higher reliability value for the online CES-D of 0.92 was obtained by Donker et al.^38^ It should be noted, however, that these authors utilised the Dutch version of the CES-D. With regard to the sensitivity and specificity of the CES-D, Donker et al.^38^ reported a sensitivity of 0.94 and specificity of 0.62 at a cut-off of 22. The authors highlighted a higher sensitivity and lower specificity of the online version of the CES-D when compared to the paper-based version of the tool.

Two studies utilised the BDI-II as an online depression screening tool. The study conducted by Holländare et al.^30^ compared the paper- and online psychometric properties of the BDI-II. The authors reported that the Cronbach’s alpha levels were similar to the online version, ranging from 0.87 to 0.89, and the paper version, ranging from 0.89 to 0.90. The participants were required to be fluent in Swedish, therefore suggesting that the BDI-II was translated. Lee et al.^34^ utilised the Korean version of the BDI-II. They did not report any online psychometric properties, but rather made reference to past studies which were paper-based psychometric properties.

Studies which utilised less common depression screening tools have reported some psychometric properties of these tools, with the exception of studies conducted by Krog et al.^28^, Leykin et al.^36^ and Liu et al.^45^ Lin et al.^39^ provided a test–retest reliability for the ISP-D of 0.83 for a 2-week period, 0.45 for a period of 2–4 weeks and, lastly, 0.50 for a period longer than 4 weeks. In addition, the authors reported a sensitivity of 76.4% and a specificity of 81.8% for 55 participants.

For the Dutch version of the SID, Donker et al.^38^ reported a sensitivity of 0.87 and specificity of 0.51 at a cut-off score of 5, as well as a 0.90 reliability for the K10 and a sensitivity of 0.69–0.81 and a specificity of 0.67–0.79 for cut-off scores of 29, 31 and 32 for the K10. When looking at the online version reliability of the MADRS-S, Holländare et al.^30^ reported that the Cronbach’s alpha of the online version was similar to that of the paper version.

The EDS evidenced a good internal consistency (*α* = 0.87) and displayed a significant correlation when compared to the online version of the BDI-II.^29^

### Applicability for South African population

Based on the review, there were no studies of online MDD screening tools targeting the South African population. It should be acknowledged that a small proportion of the sample in the study by Leykin et al.^36^ reported being South African upon follow-up assessments. Of the tools and studies identified, the majority of the tools^13^ were based on the DSM-IV classification of depression. The only tools that the researcher managed to locate in the South African context were two online questionnaires currently available on a non governmental organisation (NGO) website (see http://sadag.org/images/pdf/sphere_questionnaire.pdf and http://sadag.org/index.php?option=com_content&view=article&id=1877&Itemid=142). The NGO uses the Somatic and Psychological Health Report (SPHERE) questionnaire and the Zung Self-Rating Depression Scale. There are no published psychometric properties for either instrument for the South African population.

## Discussion

This systematic review aimed to determine if there is an appropriate online depression screening tool for use in the South African context, either in the primary healthcare sector or by the general public. Of the 17 articles included in the study, only seven articles reported psychometric properties for an online-based depression screening tool. In addition, only one article out of the 17 articles targeted a global Internet population when screening for depression. The remainder of the studies targeted very specific populations (college students, primary care patients as well as individuals diagnosed with depression).

The most commonly used online depression screening tools were the PHQ-9, CES-D and BDI-II. These results are in accordance with the most common paper-based depression screening tools, as noted by Smarr et al.^5^ In addition, despite being one of the most commonly used paper and online depression screening tools, only one article reported psychometric properties for the PHQ-9 on the general population.

The CES-D and BDI-II have been validated and compared to the paper-based version of the tool. As described by Buchanan,^20^ it cannot be taken for granted that the properties for the online and paper versions of an assessment tool are the same. However, contradictory to Buchanan,^20^ reliability properties comparison of results of the CES-D and the BDI-II online versus paper versions of these tools highlight very similar, if not the same, psychometric properties. However, when looking at the validity properties of the CES-D, it should be noted that the sensitivity is elevated and the specificity values are lowered significantly for the online version of the tool^31^ when compared to the paper-based version.^10^ These results could possibly be attributed to authors having selected a very specific target population when validating these screening tools, therefore highlighting a need for an online depression screening tool to be designed with a specific target population in mind.

The screening tools that were not among the commonly utilised depression screening tools showed relatively high psychometric properties; however, one should again be mindful of the fact that these tests were targeted to a very specific population and these results cannot be generalised to the general population. Finally, all of the screening tools utilised were based on the DSM-IV criteria and the articles included no discussion on the cross-cultural utility of the instruments used.

## Conclusion

From the study results it is evident that a space exists for an online depression screening tool, specifically for the South African context. Given the more than adequate psychometric properties exhibited by the tools, it is recommended that the online screening tool should be adapted from the three most commonly used tools: PHQ-9, CES-D and BDI-II. The items in these tools will have to be assessed for cross-cultural applicability and linguistic appropriateness. Furthermore, the ethics of online screening for MDD will have to be further explored together with issues around the accuracy and privacy of individual outcomes on online MDD screening. Given the accessibility of such tools to a global population, there will be a need to clearly state the intended target population of the screening tool.
